# Developing a Physiologically-Based Pharmacokinetic Model Knowledgebase in Support of Provisional Model Construction

**DOI:** 10.1371/journal.pcbi.1004495

**Published:** 2016-02-12

**Authors:** Jingtao Lu, Michael-Rock Goldsmith, Christopher M. Grulke, Daniel T. Chang, Raina D. Brooks, Jeremy A. Leonard, Martin B. Phillips, Ethan D. Hypes, Matthew J. Fair, Rogelio Tornero-Velez, Jeffre Johnson, Curtis C. Dary, Yu-Mei Tan

**Affiliations:** 1 Oak Ridge Institute for Science and Education, Oak Ridge, Tennessee, United States of America; 2 National Exposure Research Laboratory, US-Environmental Protection Agency, Research Triangle Park, North Carolina, United States of America; 3 Department of Environmental Engineering, North Carolina State University, Raleigh, North Carolina, United States of America; 4 Department of Physics and Physical Oceanography, University of North Carolina at Wilmington, Wilmington, North Carolina, United States of America; 5 National Exposure Research Laboratory, US-Environmental Protection Agency, Las Vegas, Nevada, United States of America; Mount Sinai School of Medicine, UNITED STATES

## Abstract

Developing physiologically-based pharmacokinetic (PBPK) models for chemicals can be resource-intensive, as neither chemical-specific parameters nor *in vivo* pharmacokinetic data are easily available for model construction. Previously developed, well-parameterized, and thoroughly-vetted models can be a great resource for the construction of models pertaining to new chemicals. A PBPK knowledgebase was compiled and developed from existing PBPK-related articles and used to develop new models. From 2,039 PBPK-related articles published between 1977 and 2013, 307 unique chemicals were identified for use as the basis of our knowledgebase. Keywords related to species, gender, developmental stages, and organs were analyzed from the articles within the PBPK knowledgebase. A correlation matrix of the 307 chemicals in the PBPK knowledgebase was calculated based on pharmacokinetic-relevant molecular descriptors. Chemicals in the PBPK knowledgebase were ranked based on their correlation toward ethylbenzene and gefitinib. Next, multiple chemicals were selected to represent exact matches, close analogues, or non-analogues of the target case study chemicals. Parameters, equations, or experimental data relevant to existing models for these chemicals and their analogues were used to construct new models, and model predictions were compared to observed values. This compiled knowledgebase provides a chemical structure-based approach for identifying PBPK models relevant to other chemical entities. Using suitable correlation metrics, we demonstrated that models of chemical analogues in the PBPK knowledgebase can guide the construction of PBPK models for other chemicals.

## Author Summary

Physiologically-based pharmacokinetic (PBPK) models are complex kinetic models describing the absorption, distribution, metabolism and excretion of chemicals in humans or animals *in vivo*. They can be utilized in many applications, such as dosimetry testing, toxicological investigations, and chemical risk assessment. *De novo* construction of PBPK models can be very challenging when chemical data are limited. Previously developed PBPK models from structurally similar chemicals can provide valuable insight in the construction of a new model. We compiled a PBPK knowledgebase that contains the chemical space covered by existing PBPK models. This knowledgebase indexes PBPK publications with chemical names, animal species, routes of administration, and model compartments. The knowledgebase aids new PBPK model generation by providing a structure-based approach to identify literatures related to provisional nearest-neighbor chemicals. Such approaches can complement efforts to develop *de novo* PBPK models that might act as supporting computational tools in modern risk assessment.

## Introduction

Developing physiologically-based pharmacokinetic (PBPK) models for chemicals can be resource-intensive, as the formulation of new PBPK models is dictated by multiple factors. These factors include intended use for the model, target organism, target subpopulation (e.g., life stage, gender), endpoint of interest (which can affect which organs are modeled individually and which are lumped together), routes of exposure, dosing regimen or exposure scenarios, and availability of relevant data for model calibration or evaluation. Among these factors, collecting chemical-specific data for parameterizing and calibrating a PBPK model is often the most resource-intensive task. For example, tissue-blood partition coefficients, or data that can be used to estimate these coefficients (e.g., log *K*_OW_), can be missing. Several *in silico* models are available for predicting tissue-specific partition coefficients based on chemical structure or properties [1–7]. On the other hand, computational tools for predicting a chemical’s metabolic pathway and rates of metabolism have been more difficult to develop due to widely variable interspecies (e.g., rat vs. human), intraspecies/interindividual (i.e., fast vs. slow metabolizers), and intra-individual (i.e., liver vs. kidney) variation in metabolic activities [8]. Many environmental chemicals and most pharmaceuticals are metabolized in the body, and metabolites often exhibit drastically different pharmacokinetic properties and toxic effects than the parent compound or alternative metabolites of the parent compound [9]. While progress has been made toward increasing the accuracy of *in silico* predictions of metabolic parameters [10–12], experimental data is always preferable, but much more costly to obtain. In addition to a dearth of chemical-specific data, time course measurements of tissue concentrations, along with dose-response measurements that reflect the disposition of a chemical and its metabolites inside the body, often do not exist, further impeding model validation.

Chemical-specific parameters or *in vivo* pharmacokinetic data are unavailable for the vast majority of chemicals in commerce. Previously published PBPK articles are great resources to search for well-parameterized and thoroughly-vetted models that can inspire the structural design, code implementation, parameter optimization and experimental validation of models for additional chemicals. Incremental improvements, adaptations or modifications of existing models are common strategies used in the PBPK field to extrapolate chemical effects from laboratory animals to humans [13–17], to incorporate additional exposure routes or life stages [18–22], to link to pharmacodynamic endpoints [23–27], or to build new models for similar chemicals [28–33].

While adapting a model to use for a different chemical has been demonstrated previously [4,34–41], the actual process of selecting the most suitable published PBPK model for use as a starting template is not trivial. One strategy is to identify existing PBPK models that describe chemical analogues of the chemical entity of interest. This approach also works when adapting only a portion of the model (i.e., its compartmental structure) for chemicals that are similar to previously modeled chemicals [42–44]. A simple, yet efficient way to identify analogous chemicals is by conducting a similarity search in a comprehensive knowledgebase. Many online tools, such as PubChem (https://pubchem.ncbi.nlm.nih.gov/) and ChemSpider (http://www.chemspider.com/) provide similarity-searching capabilities on generic sets of chemicals, but currently no repository exists specifically for PBPK models. Thus, the objective of this study is to compile a knowledgebase that contains PBPK modeling-related literature annotated with respective chemical structures along with several easily accessible molecular descriptors for these chemicals. These molecular descriptors can then be used to build a correlation matrix for each unique chemical in the knowledgebase. The knowledgebase can be queried by inputting the structure of the chemical of interest so that existing PBPK-related literature containing that chemical’s close analogues might be found. Illustration of this approach involved two case studies. In the first, the PBPK knowledgebase and correlation matrix were applied in the development of a new PBPK model for ethylbenzene using parameter values from six chemicals. These new models were then evaluated by comparing model-simulated blood concentrations of ethylbenzene against measured literature values. In the second case study, a published model of gefitinib was used to predict blood concentrations of its close-analogues and non-analogues categorized using the PBPK knowledgebase and correlation matrix. In addition to enhancing the efficiency of analogue-based PBPK model construction for additional chemicals, the power of the PBPK knowledgebase lies in its compilation of a wealth of information related to these PBPK chemicals, such as time course tissue concentration data, dose-response data, the authors’ assumptions about the model, limitations and applications of the model, and cited material. The PBPK knowledgebase directs users to published knowledge describing a specific chemical in order to aid in the development of new PBPK models for additional chemicals of interest.

## Methods

### Development of the PBPK knowledgebase

A compilation of all Supplementary Tables from the current study were summarized in a separate web repository in csv format (https://sites.google.com/site/pbpkknowledgebase/supplementary-materials). An open-source web interface is currently under development to provide intuitive navigation to data of interest for users.

### Creation of an abstract-based PBPK corpus

An abstract-based PBPK corpus was created to provide a comprehensive composition of PBPK-related literature using PubMed (http://www.ncbi.nlm.nih.gov/pubmed). Query parameters included: “pbpk OR (“physiologically based” AND (pharmacokinetic OR toxicokinetic))”.

URL: http://www.ncbi.nlm.nih.gov/pubmed/?term=pbpk+OR+(%22physiologically+based%22+AND+(pharmacokinetic+OR+toxicokinetic))

Additional search filters included “Abstract/title only.” No publication date boundaries were set for the query. Search results returned articles that were available only as early as 1977. All search results were saved and exported as a text file (S1 Table).

### Extraction of chemical names from the abstract corpus and formation of the PBPK knowledgebase

The PBPK abstract corpus (as a text file) was loaded into Google sites to be processed through www.chemicalize.org (developed by ChemAxon), which is a public web resource that uses chemical named-entity recognition (NER) and a chemical taxonomy mark-up utility to identify unique chemical structures from text. The entire corpus was subdivided into smaller sections (~400 abstracts per set) to accommodate the processing capability of chemicalize.org. The marked-up page source was copied into Microsoft Excel 2007, parsed, and filtered so that the only entries remaining were chemical names and PubMed manuscript ID (PMID) (a unique database-designated index for cataloging purposes). This process identified 795 abstracts containing specific chemical names; results are summarized elsewhere (S2 Table). Because many chemicals have more than one abstract associated with each chemical name, the CAS registry number and SMILES string for these chemicals were obtained from other databases (e.g., ACToR [http://actor.epa.gov/actor/faces/ACToRHome.jsp), DSSTox [http://www.epa.gov/ncct/dsstox/], and ChemSpider [http://www.chemspider.com/]). Duplicates and synonyms were removed based on the CAS registry number and SMILES strings. For quality control purposes, two authors manually curated the chemical list to ensure that the knowledgebase contains only specific chemical entities (e.g., “ethyl” was excluded) and that PBPK models exist for these chemicals (e.g., existing studies measuring kinetic data for a specific chemical that could be used to build a PBPK model). After the manual curation, 307 unique chemicals remained. Their chemical names, CAS registry numbers and SMILES strings are provided elsewhere (S3 Table). The 795 abstracts (S2 Table) and corresponding 307 unique chemicals (S3 Table) are referred to as the “**PBPK knowledgebase**” throughout this article.

### Mining the knowledgebase for PBPK-related terms and binary-vector determination

The abstracts in the PBPK knowledgebase were analyzed in order to identify the presence or absence of PBPK-associated word-stems. The purpose of this analysis was to improve our knowledge of the type of PBPK model information that could be expected from a publication. The PBPK-associated word-stems selected for our analyses were as follows: Species included “rat, rats, mouse, mice, human, pig, cow, goat, guinea pig, hamster, marmoset, monkey, rabbit, rhesus, rodent, sheep, bird, chicken, fish, pony, swine, turkey, and whale.” Life stages included “adult, pregnant, children, lactating, fetus, infant, dam, neonate, pediatric, pup, child, fetal, neonatal, and maternal.” Gender included “female, male, man, woman, men, and women.” Compartmental organs included “cutaneous, venous, arterial, carcass, body, fin, skin, lungs, heart, adipose, fat, brain, kidney, liver, bone, placenta, testes, ovary, breast, hepatic, blood, urine, plasma, plasma, feces, fecal, renal, milk, and hair.” Mining for these terms in each chemical name-containing abstract was performed using the open-source statistical program R (R Foundation for Statistical Computing, Vienna, Austria) to create a presence (1) or absence (0) vector (summarized in S2 Table).

### Calculating physicochemical descriptors for compounds in the corpus

Absorption, distribution, metabolism and elimination (ADME) of chemicals are largely governed by their physicochemical properties [2,3,45–48]. For each of the chemicals identified in the PBPK abstract corpus, eight easily obtainable 2D physicochemical molecular descriptors were calculated using the proprietary software Molecular Operating Environment (MOE) (Chemical Computing Group Inc., Montreal, QC, Canada). These descriptors include molecular weight (MW), hydrogen bond acceptor count (hba), hydrogen bond donor count (hbd), number of rotatable bonds (nRotB), polar surface area or topological polar surface area (PSA), octanol:water partition coefficient (logP), log transformation of solubility (logS) and area of van der Waals surface (vdw_area). Descriptor values are summarized in S3 Table.

These descriptors are commonly accepted by the research community as correlated with chemicals’ pharmacokinetic properties. MW, hba, PSA, logP and logS have been associated with human intestinal absorption [49–51]. MW hba, hbd, nRotB and PSA can be used to predict clearance and volume of distribution [52]. MW, logP, hba, hbd, PSA, nRotB and logS have been associated with percent binding to plasma and liver microsomal proteins [53,54]. MW, hba, hbd, PSA, logP were included in the *in silico* identification of cytochrome P450 isoform-specific substrates [55,56].

Because other studies have shown that increasing the number of descriptors does not necessarily increase the predictive power from descriptor to PK properties [47,57], and to limit descriptors to those that are easily accessible to the public, no additional descriptors were calculated for this study.

### Normalizing descriptors and calculating correlation coefficients

In this study, the similarity between chemicals was calculated as correlation coefficients based on the eight descriptors described above. Since the scientific community lacks consensus on the weight of importance for each descriptor toward a chemical’s pharmacokinetic properties, each descriptor was considered to contribute equally to the calculation of correlation coefficients. Six of the eight descriptors, hba, hbd, nRotB, PSA, vdw_area and MW, have values 0 or above and are positively skewed to the right. Thus, a log transformation was conducted to normalize these descriptors. The remaining two descriptors, logP and logS, exist in their transformed states. All the log-transformed descriptors were converted to standard normal distribution ~N(0,1), based on Eq 1.

$$X_{ST\_k\_i}=\frac{X_{\_k\_i}-\mu_{\_k}}{\sigma_{\_k}}$$ (1)

Where is the normalized value of the *k*^th^ descriptor for chemical *i*, *X*__*k*_*i*_ is the value of the log-transformed *k^th^* descriptor for chemical *i*, and *μ*__*k*_ and *σ*__*k*_ are the respective mean and standard deviation values of the log-transformed *k*^th^ descriptor for all chemicals. Normalized molecular descriptors for all chemicals in the PBPK knowledgebase are summarized in S4 Table.

A correlation coefficient was then calculated based on the normalized molecular descriptors, as shown in Eq 2.

$$C_{ij}=\frac{\sum_{k=1}^{8}\left(X_{ST\_k\_i})(X_{ST\_k\_j}\right)}{\sqrt{\sum_{k=1}^{8}{\left(X_{ST\_k\_i}\right)}^{2}\sum_{k=1}^{8}{\left(X_{ST\_k\_j}\right)}^{2}}}$$ (2)

Where *C*_*ij*_ represent the correlation coefficient between chemicals *i* and *j* in the knowledgebase. *X*_*ST*_*k*_*i*_ and *X*_*ST*_*k*_*j*_ represent the normalized *k*^th^ descriptor of chemical *i* and *j*, respectively. The pairwise correlation coefficients matrix for each chemical in the PBPK knowledgebase is summarized elsewhere (S5A Table). Each cell in the matrix represents the correlation coefficient between two chemicals (column and row names). This matrix has been further flattened into chemical-pairs, and then ordered by rank based on their correlation coefficient values. The rank-ordered correlation coefficients of chemical pairs are provided in S5B Table.

### Case study with ethylbenzene

To demonstrate the utility of the PBPK knowledgebase in finding analogous chemicals with existing PBPK models that could act as a starting template to build a new model, ethylbenzene was used as a case study. Six chemicals with varying structural similarities towards ethylbenzene were selected from the PBPK knowledgebase, and the equations/parameters from their existing models were used for the construction of an ethylbenzene PBPK model. The simulation results from these newly constructed models were compared to the experimental data on ethylbenzene [29].

#### Correlation coefficient-based selection of entries in the PBPK knowledgebase

First, eight molecular descriptors for ethylbenzene were calculated in MOE and normalized as described above for the chemicals contained within the PBPK knowledgebase. Second, the correlation coefficients of ethylbenzene with all chemicals in the PBPK knowledgebase were calculated and rank ordered (S6 Table). Because the experimental data [29] for ethylbenzene were extracted from a rat inhalation study, only the PBPK knowledgebase entries that had “rats” and “inhalation” in the title and/or abstract were considered for our case study. Six chemicals were selected from three categories, including: (1) exact matches (ethylbenzene), which have a correlation coefficient of 1; (2) close-analogues (xylene, toluene and benzene), which have high-ranked correlation coefficients among chemicals in the PBPK knowledgebase; and (3) non-analogues (dichloromethane and methyl iodide), which have low-ranked correlation coefficients among chemicals in the PBPK knowledgebase. The chemical names, CAS number, correlation coefficients (toward ethylbenzene), rank of correlation coefficient (among a total of 307 chemicals in the PBPK knowledgebase) and PBPK literature references of the six selected chemicals are summarized below (Table 1).

**Table 1:** Selected analogues^*^ of ethylbenzene.

Name	CAS No.	Correlation Coefficient	Rank*	PBPK ref.
Ethylbenzene	100-41-4	1		[58]
Xylene	1330-20-7	0.999979	1	[58]
Toluene	108-88-3	0.999957	3	[58]
Benzene	71-43-2	0.999848	7	[58]
Dichloromethane	75-09-2	0.988299	54	[59]
Methyl iodide	21410-51-5	0.910114	283	[60]

*Chemical analogues were selected based on their similar published experimental designs (animal species = rats, route of administration = inhalation, time span = short term) relative to that of ethylbenzene-associated literature.

*The ranking of correlation coefficient among a total of 307 unique chemicals in the PBPK knowledgebase.

#### Using newly developed models to simulate ethylbenzene blood concentrations

PBPK models for the selected entries (ethylbenzene, xylene, toluene, benzene, dichloromethane, and methyl iodide) were extracted from the literature in Table 1 [58–60]. The models were coded in MATLAB R2014b (version 8.4; The MathWorks, Natick, MA) and modified to simulate the time course of ethylbenzene blood concentrations in rats weighing 250 g after inhalation exposure to 100 ppm ethylbenzene for 4 hours. For simplicity and illustrative purposes, uncertainties in model parameters, model predictions and experimental observations were not considered: All parameters in the newly built models were set as fixed, and all data points extracted from the literature were fixed at their mean values.

The simulation results were then compared to experimental data for ethylbenzene obtained from the literature [29]. Only blood concentrations were compared for illustrative purposes. Organ-specific or tissue-specific concentration data were not measured in many studies, especially human studies, due to economic and ethical reasons. Therefore, organ-specific data were not used in the current studies.

For each of the six models, goodness-of-fit between predicted blood concentrations and measured values was calculated through the calculation of Chi Square statistics (χ^2^), using Eq 3 as follows.

$$\chi^{2}Stat=\sum_{i=1}^{k}\frac{{\left(o_{i}-e_{i}\right)}^{2}}{e_{i}}$$ (3)

Where *O*_*i*_ is the model-predicted concentration and *e*_*i*_ is the experimentally observed concentration at the *i*_*th*_ time point. *p—*Values for the χ^2^ statistics were obtained through MS Excel function “CHIDIST”. Calculated χ^2^ statistics and *p-*values for each model versus experimental data are stored in S7 Table.

#### Comparing model parameters

Parameters from the existing models (Table 1) for ethylbenzene, xylene, toluene, benzene, dichloromethane, and methyl iodide were extracted from published studies associated with abstracts contained within the PBPK knowledgebase [58–60]. These parameters can be grouped into two general types: physiology-specific and chemical-specific. Rat physiology-specific parameters (e.g., cardiac output, alveolar ventilation rate, tissue volumes, blood flows to tissues) from these models were generally consistent [58–60], while chemical-specific parameters varied widely among models (Table 2).

**Table 2:** Partition coefficients and metabolic parameters from existing PBPK models for ethylbenzene and selected analogues.

Parameters	Ethyl Benzene	Xylene	Toluene	Benzene	Dichloromethane	Methyl iodide
**Blood:air**	42.7	46	18	15	19.2	39
**Fat:air**	1556	1859	1021	500	120	89
**Slowly perfused tissue:air**	26	41.9	27.7	15	7.9	7.5
**Rapidly perfused tissue:air**	60.3	90.9	83.6	17	14.2	9
**Liver:air**	83.8	90.9	83.6	17	14.2	24
**Maximum rate of metabolism (mg/h/kg)**	6.39	6.49	3.44	2.11	4.3	831
**Michaelis-Menten constant (mg/L)**	1.04	0.45	0.13	0.01	0.4	3.6

### Case study with gefitinib

Models for the anti-cancer drug gefitinib [61] were coded and executed in Matlab in order to predict blood concentrations for seven other chemicals of varying similarity to gefitinib. Predicted blood concentrations for these seven structural analogues were then compared against measured values [26,61–67]. All entries in the PBPK knowledgebase were first ranked based on their similarity toward gefitinib, as described above (S8 Table). Close- and non-analogues of gefitinib were selected from the top and bottom of the ranking list. Because some of the top or bottom ranked chemicals do not have published experimental data, only entries that are associated with experimental data were kept as examples. Four close-analogues (itraconazole, cocaine, diclofenac and 3,3'-diindolylmethane) and 3 non-analogues (perchlorate, phosphorothioate oligonucleotide, and melamine) of gefitinib were selected for experimental data extraction (Table 3). The existing gefitinib model [61] was used to simulate the blood concentrations for these selected example chemicals. Dose and body weight (BW) were obtained from references for each chemical [26,61–67]. The volume of distribution (V1, V2) for each new chemical was linearly scaled by body weight. For example, V1__itraconazole_ = (BW__itraconazole_ /BW__ gefitinib_)* V1__ gefitinib_. Other parameters were not altered from the original gefitinib model (Table 4). Predicted blood concentrations were then compared to published experimental concentrations for each chemical. Calculation of Chi Square statistics (χ^2^) as an indication of goodness-of-fit was performed as described above.

**Table 3:** Chemical names, CAS numbers, rank of correlation coefficient toward gefitinib, and related references of selected analogues of gefitinib.

Name	CAS No.	Rank	Species	Route of Administration	PBPK ref.
gefitinib	184475-35-2	1	mice	oral	[61]
itraconazole	84625-61-6	2	human	oral	[62]
cocaine	50-36-2	5	human	intravenous	[63]
diclofenac	15307-86-5	7	human	oral	[64]
3,3'-diindolylmethane	1968-05-4*	8	mice	oral	[65]
perchlorate	14797-73-0	301	rat	intravenous	[26]
phosphorothioate oligonucleotide	10101-88-9	302	rat	intravenous	[66]
melamine	108-78-1	304	pig	intravenous	[67]

**Table 4:** Model parameters for selected gefitinib’s close-analogues and non-analogues.

Name	Dose (mg/kg)	BW (g)	Bioavailability	Absorption rate (1/h)	Elimination rate (1/h)	High- to low- permeability tissue rate (1/h)	Low- to high- permeability tissue rate (1/h)	High- permeability tissue volume (V1) (ml)	Low permeability tissue volume (V2) (ml)
gefitinib	55	22.5	0.45	0.88	0.54	1.65	0.55	75	0.3
itraconazole	2.9	70000	0.45	0.88	0.54	1.65	0.55	233330	933
cocaine	0.3	70000	0.45	0.88	0.54	1.65	0.55	233330	933
diclofenac	0.7	70000	0.45	0.88	0.54	1.65	0.55	233330	933
3,3'-diindolylmethane	250	25	0.45	0.88	0.54	1.65	0.55	83	0.33
perchlorate	3.3	330	0.45	0.88	0.54	1.65	0.55	1100	4.4
phosphorothioate oligonucleotide	10	250	0.45	0.88	0.54	1.65	0.55	833	3.3
melamine	6.13	100000	0.45	0.88	0.54	1.65	0.55	333333	1333

## Results

### Trends in PBPK-related literature

**Fig 1:**
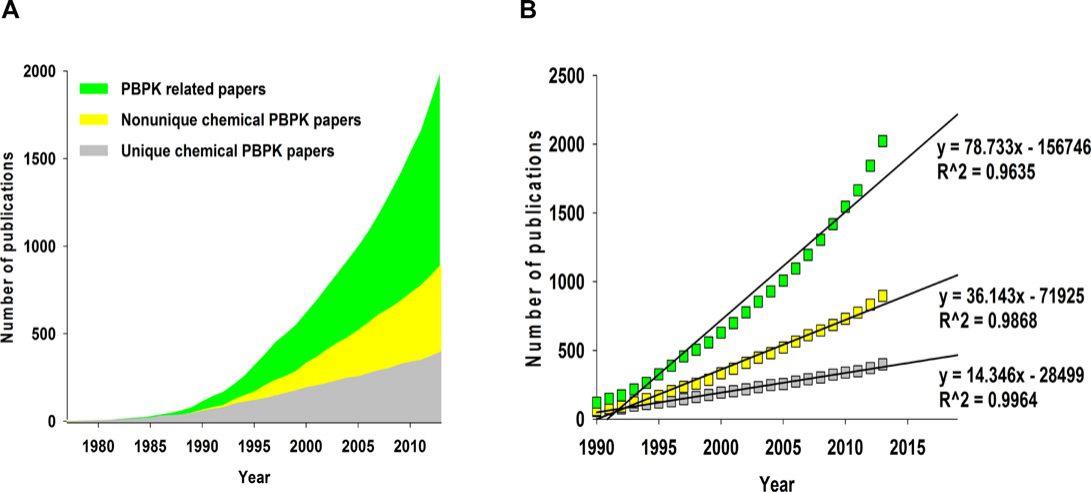
Trends of PBPK literatures. (A) The 2,039 PBPK-related articles are placed into one of three categories: (1) unique chemical PBPK papers (grey), pioneering articles in which specific chemical names have appeared for the first time; (2) non-unique chemical PBPK papers (yellow), articles in which chemical names have appeared in previous publications; or (3) PBPK related papers (green), articles that are not associated with specific chemical names. (B) Linear regression of the number of articles in three categories over time.

The 2,039 PBPK-related articles were assigned to one of three categories (Fig 1A): publications on unique chemicals that appeared for the first time (likely to be a newly-developed PBPK model); publications on chemicals that appeared in previous publications (likely to be an application or refinement of a previously-developed PBPK model); and reports on general PBPK concepts, methods, commentaries, perspectives, or reviews.

Regression analysis was performed for the three categories (Fig 1B). Linear relationships between the number of publications and the year of publication were calculated to help identifying the growth rates. The growth rates for publications are 14/year, 36/year, and 78/year for the first, second, and third categories, respectively. These trends reflect the difficulty in developing a new PBPK model due to the great quantity of experimental data required. While our search suggests ongoing development and expansion of PBPK-related modeling methodologies, the low output of new PBPK models limits the utility of PBPK modeling for examination of health risks resulting from chemical exposures.

### Species, life stage, gender, and organ coverage of the PBPK knowledgebase

**Fig 2:**
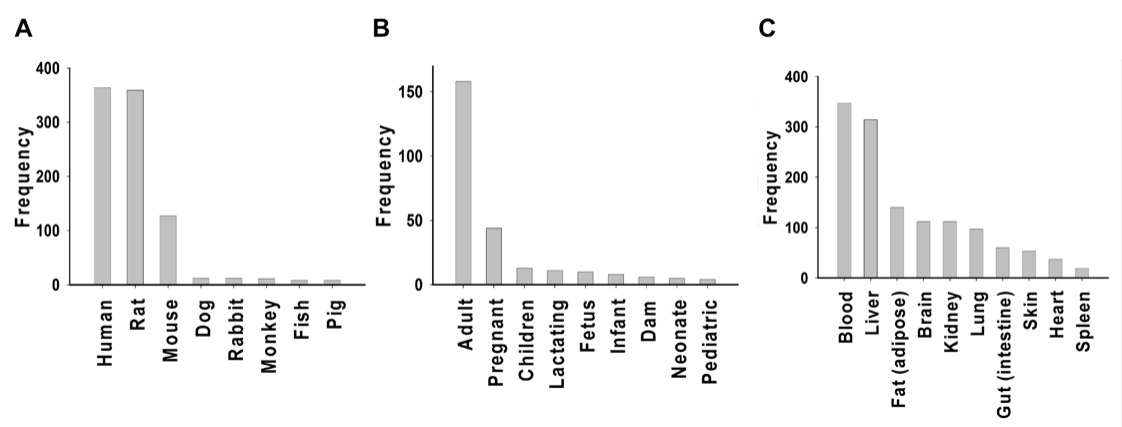
Keywords extraction from PBPK literatures. The abstracts in the PBPK knowledgebase were analyzed to identify PBPK-associated word-stems: (A) Frequency of the top 10 species; (B) Frequency of the top 10 life stages; (C) Frequency of the top 10 compartments.

When comparing the two gender keywords, “male” appeared much more frequently than “female” (66% vs. 34%). “Human” and “rat” were the most frequently mentioned species, and these key words appeared at about three times the frequency of “mouse” (Fig 2A). “Dog,” “rabbit,” “monkey,” “fish,” and “pig” comprised the 4^th^ to 8^th^ most common animal species mentioned in the abstracts, but with noticeably lower frequency than the top 3 species, which accounted for >94% of the total (Fig 2A).

The most frequently mentioned life stage was “adult,” appearing three times more frequently than the second-most frequent term “pregnant” (Fig 2B). For the top 9 life stage terms mentioned in PBPK-related literature, the key words “pregnant,” “dam,” and “lactating” refer to the reproductive cycle of the female parent; the key words “fetus,” “neonate,” “infant,”“pediatric,” and “children” refer to growth and developmental stages of offspring (Fig 2B).

“Blood” and “liver” were the two most frequent organs incorporated into PBPK models, with appearance frequencies twice as high as the 3^rd^ most frequent organ, “fat (adipose)” (Fig 2C). “Brain,” “kidney,” “lungs,” “gut (intestine),” “skin,” “heart,” and “spleen” comprised the 4^th^ to 10^th^ most frequent organs mentioned in these publications.

### Calculation of physicochemical molecular descriptors and correlation coefficients

**Fig 3:**
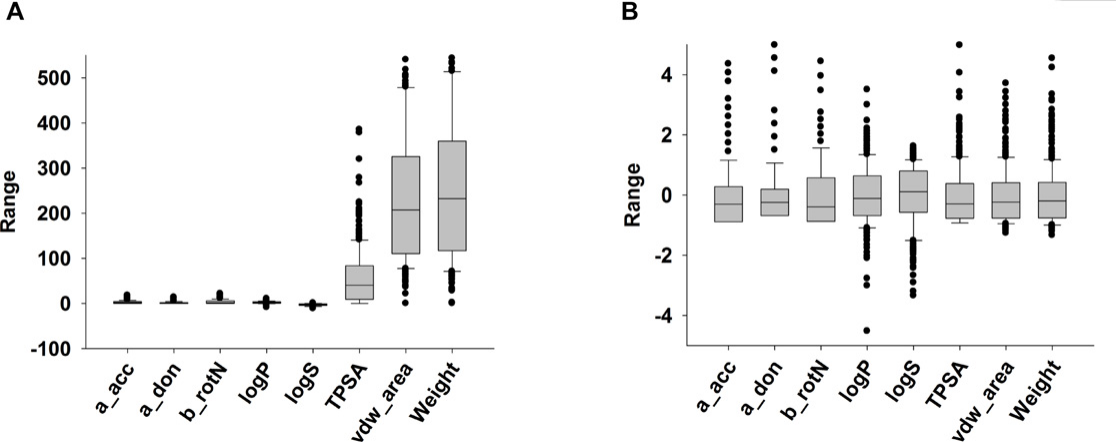
Physicochemical molecular descriptors. Summary of the values of eight physicochemical molecular descriptors, calculated using the Molecular Operating Environment (MOE), for 307 chemicals in the PBPK knowledgebase. The eight descriptors are molecular weight (MW), hydrogen bond acceptor count (hba), hydrogen bond donor count (hbd), number of rotatable bonds (nRotB), polar surface area or topological polar surface area (PSA), octanol:water partition coefficient (LogP), log transformation of solubility (logS), and area of van der Waal surface (vdw_area). (A) The original calculated descriptor values; (B) The normalized descriptor values using Eq 1 from the Methods section.

The means and standard deviations of hba, hbd, nRotB, logP and logS were much smaller than those of PSA, vdw_area and MW (Fig 3A). After normalization of the physicochemical molecular descriptors for these chemicals using Eq 1 above to reduce bias in calculation of correlation coefficients, the mean and standard deviation of each descriptor was set as 0 and 1, respectively (Fig 3B).

Each cell in the correlation matrix contains the correlation coefficient of one chemical toward another chemical in the knowledgebase. The top five correlation coefficients were equal to 1, because those five chemical pairs contained identical molecular descriptors. These chemicals were either chiral isomers or isotopically-labeled compounds. The remaining chemical-pair combinations exhibited a maximum correlation coefficient of 0.999990409 (between 1,2,4-trimethylbenzene and 1,2,3,5-tetramethylbenzene) and a minimum correlation coefficient of 0.589788342 (between ethylene and methyl mercury).

### Case study with ethylbenzene

**Fig 4:**
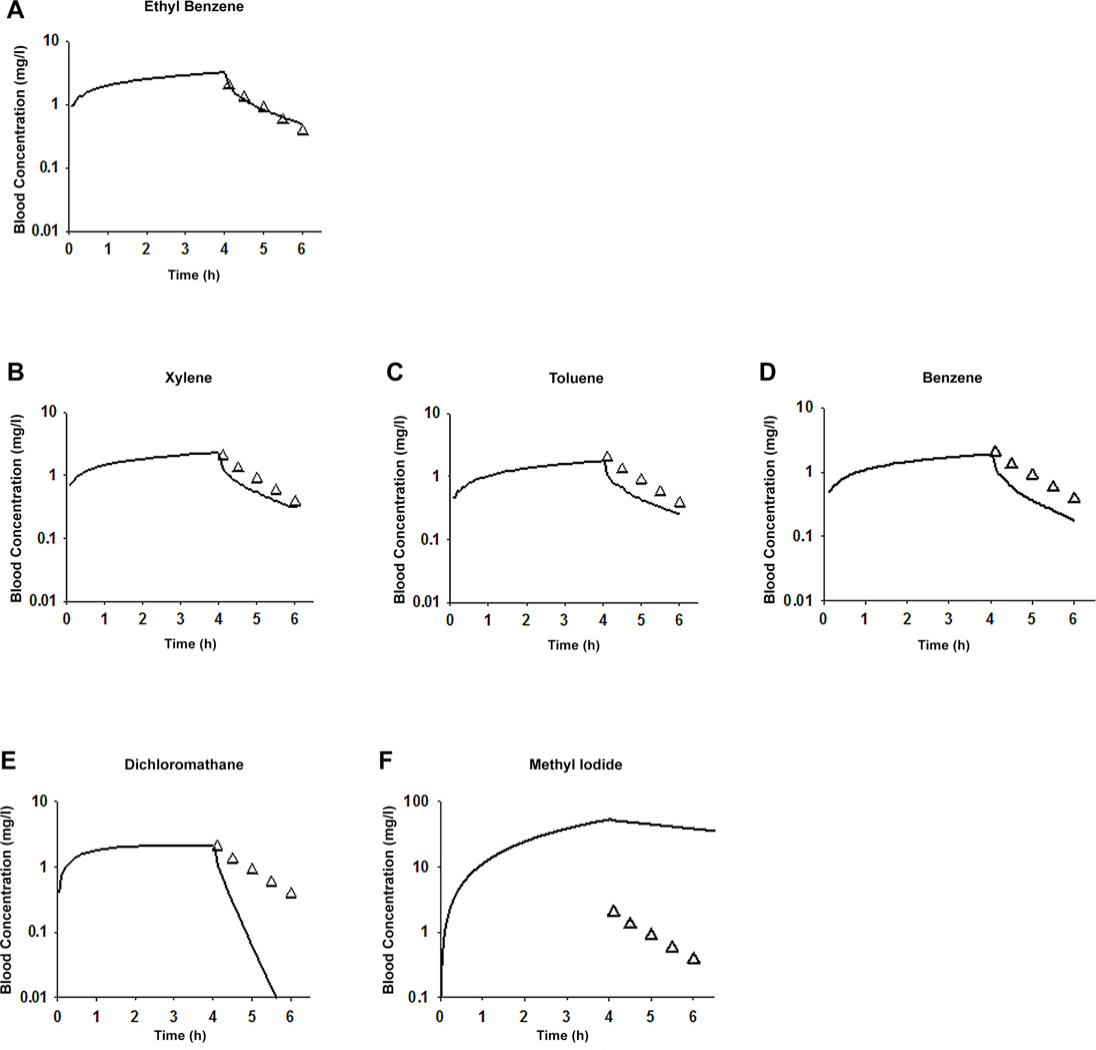
Case study with ethylbenzene. Comparing blood concentrations of ethylbenzene (triangle symbols) from rats exposed to 100 ppm ethylbenzene for four hours [29] and simulated blood concentrations of ethylbenzene (solid lines) based on the (A) ethylbenzene PBPK model [58]; (B) xylene PBPK model [58]; (C) toluene PBPK model [58]; (D) benzene PBPK model [58]; (E) dichloromethane PBPK model [59]; and (F) methyl iodide PBPK model [60].

Simulated blood concentrations from the PBPK model based on an “exact match” (ethylbenzene) [58] aligned extremely well with the experimental data [29] (Fig 4A). Simulated blood concentrations from PBPK models based on “close-analogues” (xylene, toluene and benzene) [58] deviated slightly from the ethylbenzene data (Fig 4B and 4C and 4D). In contrast, simulated blood concentrations from the models based on “non-analogues” (dichloromethane and methyl iodide) [59,60] exhibited significant deviations from the experimental data on ethylbenzene (Fig 4E and 4F).

The PBPK model based on an “exact match” (ehtylbenzene) resulted in the highest χ^2^ goodness-of-fit *p*-value of 0.9991; PBPK models based on “close-analogues” had *p*-values of 0.8603, 0.5789 and 0.1479 for xylene, toluene, and benzene, respectively; PBPK models based on “non-analogues” (dichloromethane and methyl iodide) resulted in much lower *p*-values of <6 × 10^−215^.

### Case study with gefitinib

**Fig 5:**
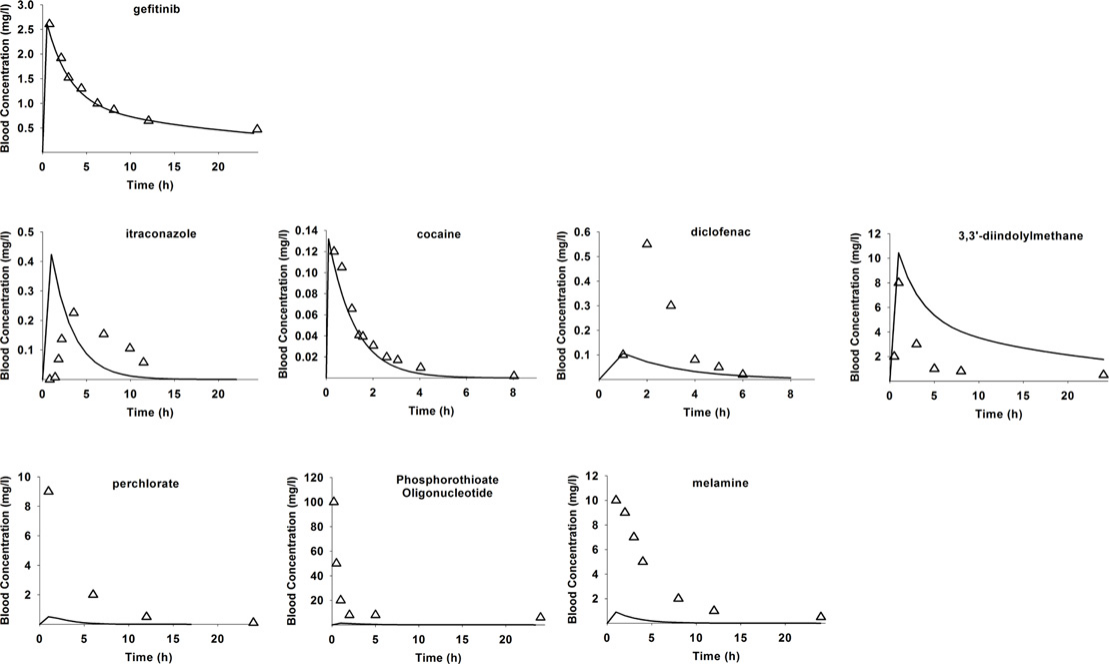
Case study with gefitinib. Comparing simulated (solid lines) and experimentally observed (triangle symbols) blood concentrations for compounds. PBPK models were extracted from the gefitinib study [61], and executed to predict pharmacokinetics of gefitinib’s close-analogues (itraconazole, cocaine, diclofenac, 3,3'-diindolylmethane) and non-analoguse (perchlorate, phosphorothioate oligonucleotide, melamine, carbamateon). The experimental observations were extracted from PBPK literature listed in Table 3.

Fig 5 shows published experimental observed blood concentrations for each example chemicals compared to their predicted values from the each of the accommodated gefitinib models (dose, BW, V1,V2 adjusted). Chi Square statistics (χ^2^) were calculated and stored in S9 Table. The gefitinib model fit the best with its own experimental data, with χ^2^ test *p*-values equal to 0.999. The predictive ability of the gefitinib model for the structural analogues cocaine and 3,3'-diindolylmethane were high, with χ^2^ goodness-of-fit *p*-values of 0.994 and 0.898, respectively. The χ^2^ test *p*-values for the other two structural analogues itraconazole and diclofenac were not as high, but still better than those of non-analogues, with χ^2^ test *p*-values of 2.81 × 10^−21^ and 5.71 × 10^−14^, respectively. The χ^2^ test *p*-values for non-analogues were all zero.

## Discussion

Researchers have used molecular modeling approaches (e.g., quantitative structure-activity relationships) to predict parameters, such as volume of distribution and clearance rate, to fill data gaps when building new PBPK models for chemicals lacking these data [52,68–70]. This approach can be labor intensive and requires background knowledge in computational chemistry and statistics. Utilizing pre-existing models with well-calibrated parameters to help new model construction is a more efficient approach and has been widely implemented [28–33]. However, reviewing or sorting through publications for relevant information often would be an overwhelming task for investigators. The PBPK knowledgebase presented in this current work serves as an effective means for finding analogues whose publications contain necessary model information and/or data that can aid the construction or validation of new PBPK models for new chemicals.

One-compartment (whole body) and two-compartment (blood and the remainder of the body) models are simplest classical pharmacokinetic models. In these models, the organ-structure and mathematical equations remain the same for different chemicals, so extracting parameters from pre-existing models would be reasonable approach [61]. When a classical PK model extends beyond two compartments, grouping and integration of organs into hypothetical compartments often occurs [71]. For example, the liver might be integrated into one compartment (e.g., compartment 3 of a five-compartment model) for one chemical but integrated into an entirely different compartment (e.g., compartment 4) for another chemical. This difference in integration could lead to changes in not only chemical-specific parameters, but also in physiological parameters (e.g. compartmental volume, protein content, metabolic capacity) for compartment 3 and 4, respectively. Therefore, extraction from pre-existing models would not be an appropriate parameterization approach for high-dimension classical PK models.

Compartments in PBPK models correspond to real biological organs (tissues), so the physiological parameters of compartments (e.g., blood flow, organ volume, and protein content) are highly conserved among PBPK models [72]. Chemical-specific parameters for each organ, such as tissue:blood partition coefficients and fraction of protein binding, are related to organ structure and the compound’s physicochemical properties. These characteristics enable researchers to use parameters from an analogue’s pre-existing model for a new PBPK model of new chemicals. However, mathematical equations in PBPK models are determined on a case-by-case basis, with variation in the number of compartments, type of compartments (flow-limited or diffusion-limited), chemical-specific elimination routes, active transport of the parent chemical and/or metabolites, and other factors [73]. Therefore, borrowing parameters from other PBPK models would be a case-by-case practice and require extensive browsing and reading of relevant publications. The PBPK knowledgebase not only ranked the publications based on structural similarity (S5A Table), but also summarized much essential information, such as chemical names, organs, genders and species (S2 Table). It will help expedite selection and reading through relevant publications for locating appropriate parameter values.

The current estimate for the number of chemicals in commerce in the United States is nearly 100,000, with 500 to 1000 new chemicals being produced each year [74]. The current rate of PBPK model development (~14 chemicals/year; Fig 1B) will likely never catch up with rate of new chemical production. Just covering the 1,800 chemicals found in consumer products would take more than 100 years. The new strategy presented in this current work is designed to facilitate the generation of provisional pharmacokinetic models as chemical inventories continue to expand. The PBPK knowledgebase can be used to gauge the chemical space of existing PBPK models, as well as developing a methodology to search for existing PBPK models for structural analogues of chemicals of interest. It is up to the discretion of future investigators whether to use our proposed approach based on eight molecular descriptors to select an analogue or to use a different similarity testing method based on the intended purpose of the new model.

Identifying structural analogues is an ongoing area of research. Commonly accepted numerical measurements of chemical similarity are distance coefficients based on chemical descriptors, such as binary (0 or 1) values, indicating the absence or presence of some particular feature, topological indices, physicochemical properties (sometimes estimated using *in silico* approaches), or on/off indications for molecular fingerprints [75]. A large number of similarity calculations have been defined and used in the literature, including Euclidean [75,76], Hamming [75,76], Minkowsky [76,77], Correlation [76,78], Tanimoto [75,76], Molecular Access System (MACCS) [79,80], and Artificial Neural Network (ANN) [81,82]. Currently, there is no consensus regarding the best practices in selecting molecular descriptors and similarity calculation methods. Pearson’s correlation coefficient has previously been used in similarity calculations [76,78]. We chose this metric because it equally-weighted the descriptors and also can easily be calculated through a simple R programming language script. The eight molecular descriptors (Fig 3) presented here were selected based on their relationship with pharmacokinetic properties [47,49,52,57,83] and their ease of accessibility to the general public.

Route of administration, as well as molecular properties of chemicals, can influence chemical behavior entering into a biological system. Route of administration determines the bioavailability, peak blood/tissue concentrations (C_max_), time of peak concentrations (t_max_), biological half-life (t_1/2_), and other pharmacokinetic characteristics [84,85]. When intravenously injected, ethylbenzene is 100% bioavailable and reaches t_max_ at time 0. When exposed through oral administration, the bioavailability of ethylbenzene is influenced by metabolic degradation in gut tissue, by gut lumen bacteria, and by liver hepatocytes [86–88]. If exposed through inhalation, ethylbenzene’s bioavailability and rate of absorption is determined by the air:blood partition coefficients and gas exchange rate of the lung [89,90]. The physiological parameters of the model change with a given animal species. Although extrapolation between species is a common practice in PBPK modeling [91], the original model is more appropriate when using the same species as that used to derive the experimental data. Therefore, in our ethylbenzene case study which used model predictions comparable to measured time course data obtained from a rat inhalation study [29], “inhalation” and “rats” were used as filters for the PBPK knowledgebase before chemical selection.

The three categories of “exact match,” “close-analogue,” and “non-analogue” in our case study represent the major scenarios that can aid researchers in model development using the PBPK knowledgebase. For a given chemical, if an “exact match” entry is found, the existing model should have the best predictive capability. Although the same information may be retrievable through a PubMed search for the chemical, our PBPK knowledgebase can be used to search existing models that might have been built based on alternatives for a chemical (e.g., synonyms, chiral isomers or isotopically-labeled compounds). For example, ChemSpider lists 21 synonyms for ethylbenzene. The PBPK knowledgebase provides a more efficient and more precise solution, especially for those without a background in chemistry: any synonyms, chiral isomers or isotopically-labeled compounds would have a calculated correlation coefficient of 1 (S5B Table).

Besides being able to search for an “exact match,” the power of the PBPK knowledgebase lies in its ability to detect “close-analogues” of a chemical simply by searching for the highest correlation-ranked entries. Searching for “close-analogues” without the knowledgebase could potentially be achieved by a two-step process of (1) searching public chemical databases for structural analogues; and then (2) searching PubMed for existing PBPK models for each analogue. This two-step process, however, is unnecessarily time-consuming. Although many publications exist that contain PBPK-related models and information, this number pales in comparison to the hundreds of thousands of chemical structures found in public chemistry databases (e.g., ChemSpider, PubChem) [92]. It is more efficient to start the search of analogues found within the PBPK knowledgebase, which contains 307 entries rather than with the entire universe of chemicals. For example, in a structural-similarity search using ethylbenzene on ChemSpider, more than 10,000 results were retrieved with a Tanimoto score >99%. Xylene and toluene, which ranked 1^st^ and 3^rd^ in the PBPK knowledgebase, were not included in the top 100 of this list of chemical analogues for ethylbenzene identified in ChemSpider. Studies have shown that not all structural properties are associated with chemicals’ PK properties [2,3,45–48]. Using all available molecular descriptors, such as the structural analogue algorithm in the public chemical database, may result in a less accurate estimation of the desired PK property analogue.

The two “non-analogues” of ethylbenzene were selected from lower correlation-ranked entries in our ethylbenzene case study to confirm that the parameter values for non-analogues differ most from parameter values for “exact matches” (Table 2), and that simulations from PBPK models of non-analogues deviate most from experimental data (Fig 4E and 4F). Since experimental data for the target chemical, ethylbenzene, was available in our case study, it was straightforward for us to categorize the knowledgebase entries as “close-analogues” or “non-analogues” by comparing model predictions with data. For a chemical of interest lacking experimental data, there is no clear way to select a threshold for similarity rankings. A proposed rule of thumb is to select three to five chemicals from the first ten correlation-ranked entities, and then the “best” published model is picked from this shortlist. We caution that this recommendation is subjective, and the choice of the best model should be rooted in the quantity and applicability of the data that is available from published research to calibrate the model. For example, a model that was calibrated using time course data in multiple tissues in animals and evaluated against human data would be considered a better model than one that was calibrated using only urinary metabolite data and was not evaluated against any human data.

Our second case study with gefitinib further demonstrates the utility and versatility of the PBPK knowledgebase. A pre-existing PBPK model of gefitinib was accommodated to predict the experimental observations of other chemicals, selected from the top and bottom of similarity-ranked PBPK knowledgebase entries. The simulated drug kinetics in Fig 5 and calculated χ^2^ test *p*-values in S9 Table, demonstrated that the gefitinib model gave better predictions for the closer structure analogues (cocaine, 3,3'-diindolylmethane) than to non-analogues (perchlorate, phosphorothioate oligonucleotide, melamine). These results supported the theoretical assumption for using close-analogue’s existing parameters for a new chemical’s model construction [4,34]. The PBPK knowledgebase not only contains the chemical names, animal species, route of administrations, and tissue compartments (all of which were extracted and used for indexing and searching in the current manuscript), but also can facilitate the discovery of corresponding experimental data that can be easily extracted from publications. Such extracted data was used to test the appropriateness of gefitinib’s model in our second case study. These data can also serve additional needs and interests of knowledgebase users.

Commercial software packages such as SimCyp, Gastroplus or PKSim are used extensively in the pharmaceutical industry, as well as in academia, to support rapid PBPK model development [93–96]. We wish to emphasize several fundamental differences between these commercial software packages and our PBPK knowledgebase. Firstly, values of chemical-specific parameters in the knowledgebase are either measured or optimized against experimental data; while in SimCyp, Gastroplus and PKSim, chemical-specific parameters are often QSAR-based predictions. Secondly, our knowledgebase provides more information than merely model code and parameter values. Through its abstract corpus compilation, the knowledgebase also refers users to relevant articles containing time course tissue concentration data, dose-response data, the authors’ assumptions, limitations and applications of the model, and cited resources, in regards to chemicals of interest. Thirdly, our knowledgebase is free to the public and acts as a central location for abstract information relevant to chemicals of interest. Users accessing this information can contact the authors of the publications for additional information pertaining to model code or related data, while commercial software packages require licensing fees. Finally, the model structures in the published literature, which can be easily located from abstract information provided in the knowledgebase, were constructed based on the authors’ expert judgement and modeling philosophy (e.g., top-down vs. bottom-up); in SimCyp, Gastroplus, and PKsim, the model structure is primarily generic. Although a modifiable, generic model structure is easy to use, more experienced users may prefer to construct their own models based on data availability and purposes of the study.

The knowledgebase herein provides abstracts for previously published PBPK articles, beginning from 1977 onwards. With extracted information from these published articles, the knowledgebase can aid users in identifying the most relevant publications. Use of this extracted information is highly dependent on the scientific questions and problems of interest and is applicable mostly on a case-by-case basis. The two case studies presented here represent two specific circumstances addressing different scientific queries. Future users should select a strategy that meets their individual needs, based on data availability and study purpose, when using the knowledgebase.

In summary, a PBPK knowledgebase was compiled that contains a thorough documentation of the chemical space of PBPK models. This knowledgebase provides scientists with a structure-based approach to identify provisional nearest-neighbor chemicals that are described by existing PBPK models and whose existing models might be used as a template to construct new models for chemicals of interest. These comprehensive dataset initiatives can be coupled with *in vitro* or *in vivo* chemical and biological data curated and accessible from other sources (e.g., STITCH 4.0, CSS Dashboard, Comparative Toxicogenomics Database), or with more recent methods such as *in silico* multi-target profiling in DockScreen [97], in order to pave the way for more rapid PBPK model development. Such approaches can complement efforts to rapidly develop PBPK/PD models that are designed to act as supporting computational tools in modern risk assessment.

## Supporting Information

S1 Table 2,039 PBPK-related articles between 1977 and 2013. (TXT)

S2 Table 795 abst, corresponding chemicals, organ, species, life stages. (CSV)

S3 Table 307 unique chemical name, CAS, smiles and descriptors. (CSV)

S4 Table N 0,1 normalized descriptors. (XLSX)

S5 Table Correlation matrix and rank_ordered. (XLSX)

S6 Table Rank of chemicals based on correlation to ethylbenzene. (XLSX)

S7 Table Chi square test ethylbenzene case study. (XLSX)

S8 Table Rank of chemicals based on correlation to gefitinib. (XLSX)

S9 Table Chi square test gefitinib case study. (XLSX)
